# An unexpected case of neonatal compartment syndrome associated with congenital anomalies of kidney and urinary tract

**DOI:** 10.1515/crpm-2022-0020

**Published:** 2023-03-07

**Authors:** Laura M. Seske, Melissa Mastroianni, Keith T. Aziz, Laura W. Lewallen

**Affiliations:** Division of Neonatology, Department of Pediatrics, The Johns Hopkins School of Medicine, Chicago, IL, USA; Department of Plastic and Reconstructive Surgery, The Johns Hopkins University, Baltimore, MD, USA; Department of Orthopaedic Surgery, The Johns Hopkins University, Baltimore, MD, USA

**Keywords:** acute compartment syndrome, desquamation, ischemia, neonatal compartment syndrome

## Abstract

**Objectives:**

Neonatal compartment syndrome (NCS) occurs when increased pressure within the fasciocutaneous compartment decreases capillary perfusion, causing irreversible tissue damage from ischemia. NCS is a rare condition that requires prompt diagnosis and treatment. Diagnosing NCS is highly dependent on the examination, which can be difficult in newborns. Prompt recognition provides the best chance for good outcomes.

**Case presentation:**

We present a case of NCS diagnosed and treated based on physical examination findings. Fetal ultrasonography showed bladder distension, bilateral hydroceles, urethra dilation, and abdominal urinary ascites concerning for lower urinary tract obstruction and possible bladder rupture. At 1 h after birth, examination of the infant’s left upper extremity showed no spontaneous movement, the hand and forearm appeared dusky, and the hand had a large blister with desquamation. No pulse distal to the antecubital fossa was detected via Doppler ultrasonography. The infant was diagnosed with NCS and underwent urgent fasciotomy. The clinical appearance and perfusion of the left upper extremity gradually improved. At four months of age, the wounds were healed and the patient had full passive range of motion of the left upper extremity. Recovery of active motion is ongoing.

**Conclusions:**

The presence of blistering and desquamation should provoke suspicion for NCS. Once NCS is diagnosed, prompt intervention is necessary to reduce the risk of poor functional outcomes. This case highlights the need for increased awareness of the risk developing compartment syndrome *in utero* as part of the rare sequalae in infants with congenital anomalies of kidney and urinary tract.

## Introduction

Neonatal compartment syndrome (NCS) occurs when increased pressure within the fasciocutaneous compartment causes decreased capillary perfusion pressure and progressive tissue damage leading to a rare and potentially devastating condition that requires prompt diagnosis and intervention [1], [2], [3], [4], [5], [6]. One of the most well-known long-term sequelae of missed diagnosis or delayed treatment of compartment syndrome is Volkmann’s ischemic contracture, resulting in intrinsic muscle paralysis and an insensate hand. Volkmann’s contracture is named for the 1881 report by Richard von Volkmann describing a 16-year-old with severe deformity of the hand and wrist resulting from a tightly wrapped bandage [7]. In addition to soft-tissue contracture, NCS may cause irreversible physeal injury, leading to a shortened limb that may necessitate future reconstructive surgery or amputation [3, 8].

Although the annual incidence of acute compartment syndrome is 1–7.3 per 100,000, it remains exceedingly rare during the neonatal period [9]. The causes of acute compartment syndrome in neonates differ from those in adults, and NCS does not present with the classic clinical signs (paresthesia, paralysis, poikilothermia, pulselessness, pallor). Although NCS has several potential causes, its rarity means that most physicians have little experience diagnosing and treating it. Intrinsic causes include arterial or venous thromboses or emboli, which can occur as a result of catheterization [2, 10], and neonatal hypercoagulability [4, 8]. Perinatal infections, hematologic disorders, liver dysfunction, and other causes may lead to neonatal coagulopathy. Extrinsic causes include oligohydramnios secondary to premature rupture of membranes, wrapping of the umbilical cord, amniotic bands [4, 8], birth trauma during fetal descent or at delivery [2], in utero mechanical compression [4]. In utero mechanical compression can occur in cases of multiple gestation, uterine abnormalities, fetal macrosomia, and excessive maternal weight gain, which may occur in the setting of maternal diabetes mellitus [8]. The differential diagnoses for NCS also include cellulitis, necrotizing fasciitis, vascular injury, brachial plexus injury, and other hematologic and dermatologic conditions [3].

Prompt diagnosis and intervention of NCS have resulted in good clinical outcomes [2, 4]. In the largest single-institution case series published, a skin lesion on the forearm was present in all 24 cases of NCS [4]. We report a case of NCS associated with congenital anomalies of kidney and urinary tract diagnosed largely because of the appearance of the skin lesions on the forearm and treated within five to 6 h after birth, and we describe the early outcome of prompt surgical intervention.

## Case presentation

A Black male infant weighing 2,021 g was born at 35 weeks and three days of estimated gestational age by repeat cesarean delivery because of concern for decreased fetal movement in the setting of a low biophysical profile score (based on muscle tone, heart rate, movement, breathing, and amniotic fluid volume). Maternal history was notable for sickle cell trait, advanced maternal age, and obesity. Pregnancy history was notable for oligohydramnios observed via ultrasonography at 31 weeks. Fetal ultrasonography also showed bladder distension, bilateral hydroceles, urethra dilation, and abdominal urinary ascites concerning for lower urinary tract obstruction and possible bladder rupture. Rupture of membranes occurred at delivery.

**Figure 1:**
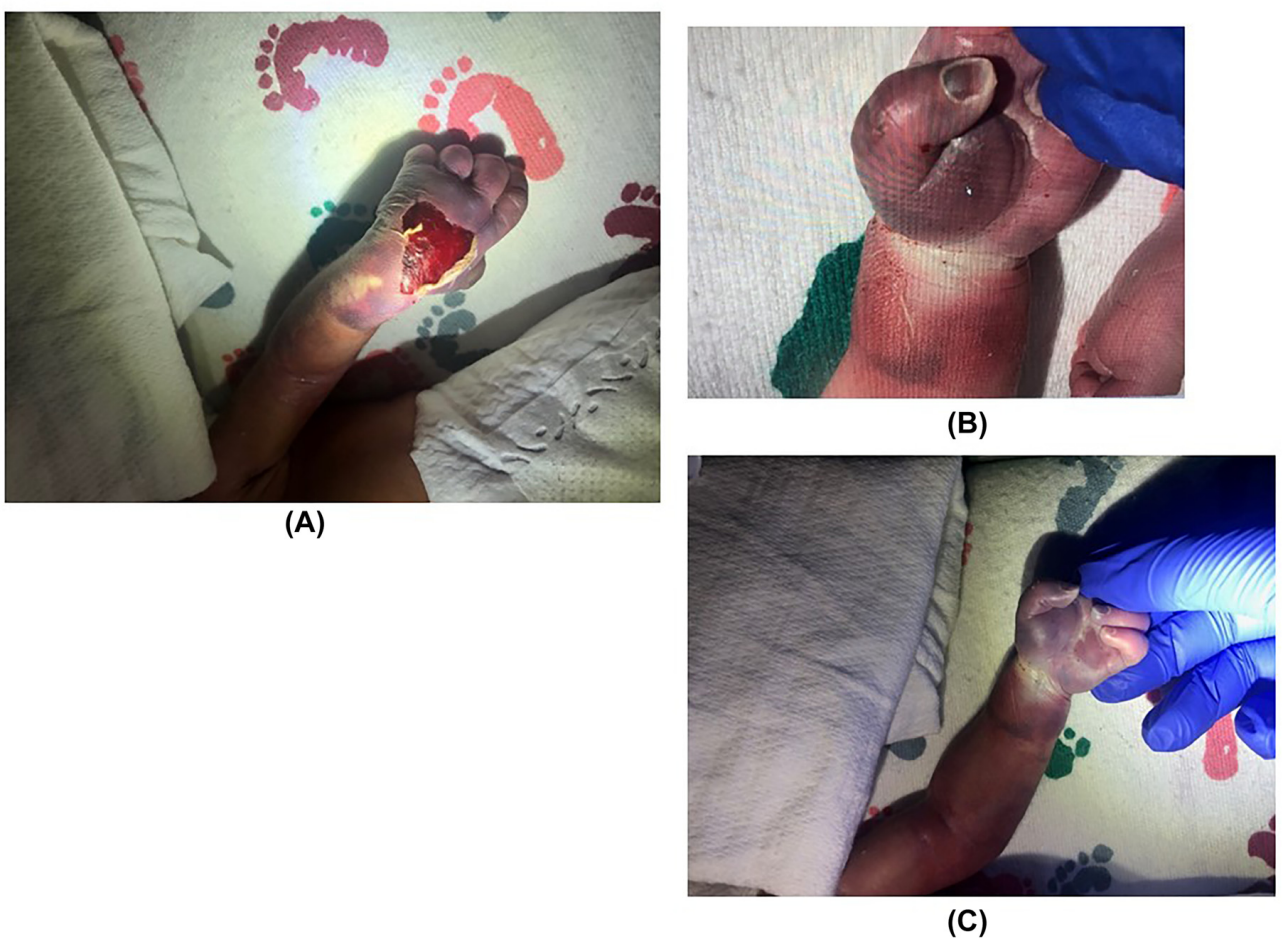
Initial examination. Appearance of the left upper extremity at 1 h of life in an African-American male infant weighing 2,021 g who was diagnosed with neonatal compartment syndrome. (A) The dorsum of the hand had a large area of desquamation. (B) The palmar aspect was cool to the touch, with dark discoloration of the thenar eminence that extended. (C) Proximal to the wrist crease to the distal third of the forearm.

At delivery, the infant experienced respiratory distress and was treated with positive pressure ventilation, supplemental oxygen, and intubation. Apgar scores were two and seven at one and 5 min of life, respectively. Examination showed substantial bruising and duskiness of the left upper extremity. The infant was transferred to the neonatal intensive care unit for treatment of respiratory distress and suspected lower urinary tract obstruction. On evaluation within 1 h after birth by orthopaedic and plastic surgery specialists, the distal third of the forearm and hand were cyanotic, and a large blister and surrounding area of desquamation were evident on the dorsum of the hand (Figure 1A–C). No spontaneous movement of the left upper extremity was observed, and no withdrawal or response to stimuli was elicited. Doppler ultrasonography showed arterial flow to the level of the antecubital fossa, but no radial or ulnar pulses were detected. Physical exam findings were consistent with NCS, likely due to compression from uterine wall due to oligohydramnios during pregnancy.

**Figure 2:**
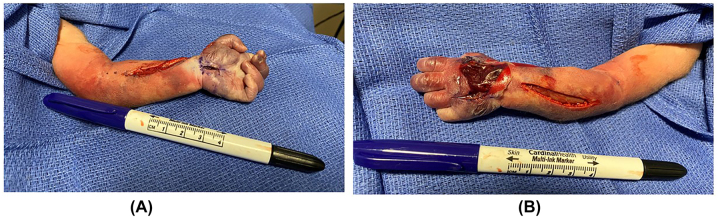
Fasciotomy. Photographs taken approximately 6 h after birth, after fasciotomies were performed on the (A) volar surface of the forearm, thenar eminence (with a carpal tunnel release), and the (B) dorsal surface of the forearm, including interosseous decompressions through two dorsal incisions.

**Figure 3:**
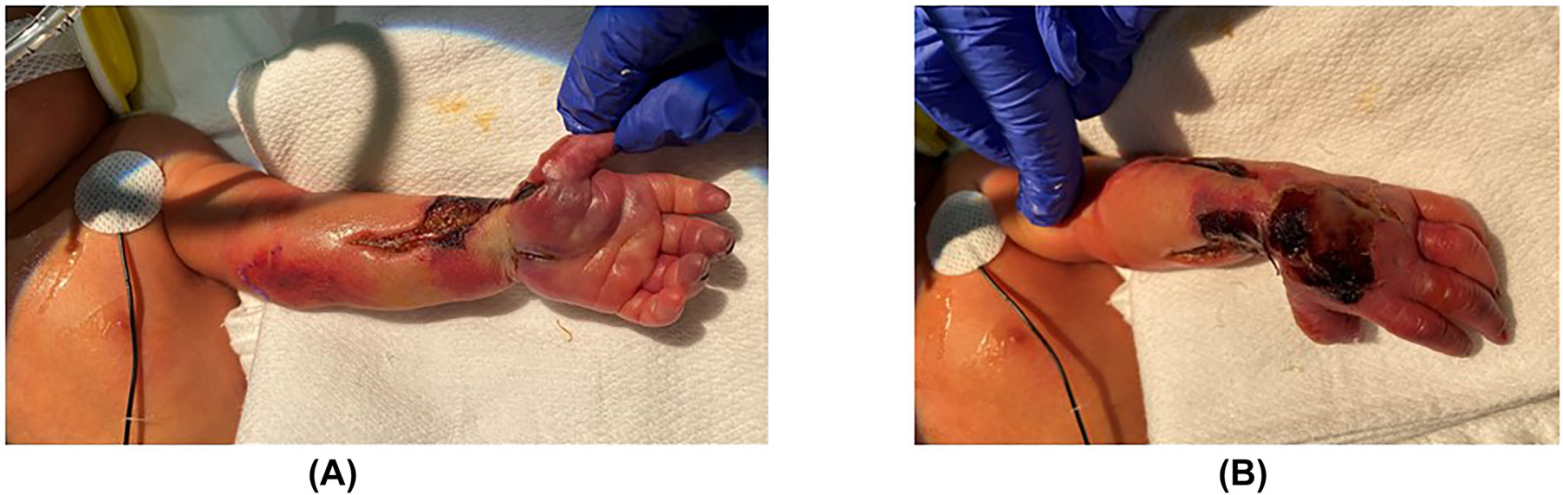
Third day of life. The fasciotomy sites were treated nonoperatively with wound care and showed no signs of infection on day three of life. (A) The volar surface healed well. (B) The dorsal surface had some areas of necrosis that were treated with mild chemical debridement agents.

**Figure 4:**
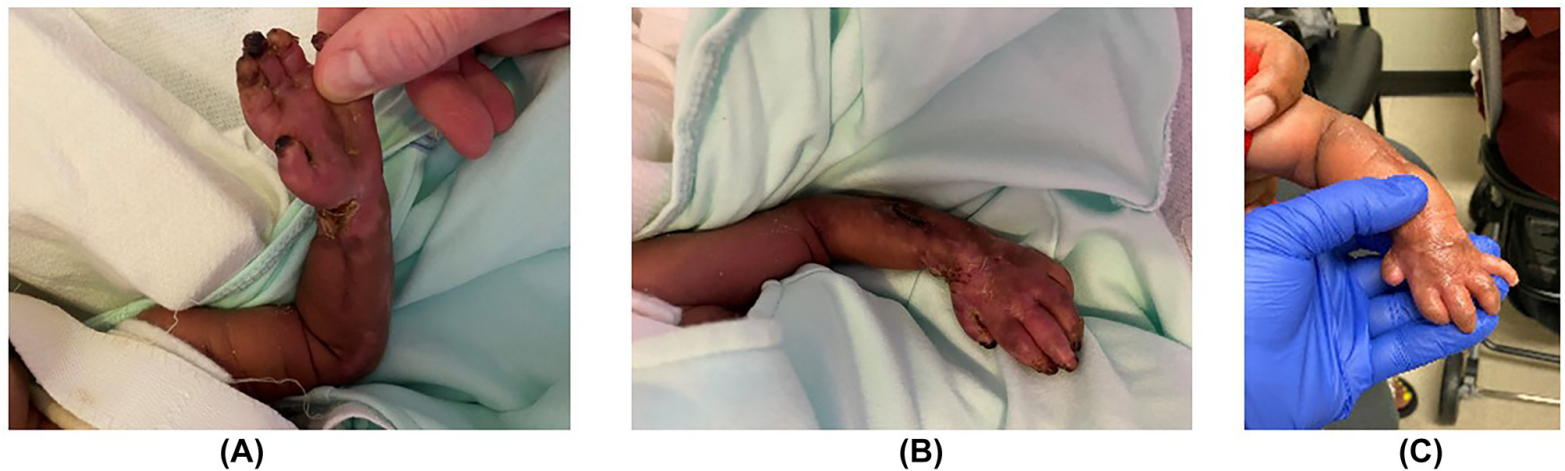
Follow-up. The wounds were completely healed at six weeks of life after nonoperative wound care. (A) Small areas of necrosis are evident at the fingertips. (B) Perfusion of the forearm improved since fasciotomies were performed. (C) Four-month follow-up. At four months of age, the patient continued to have well-healed wounds and full passive range of motion. Active range of motion continued to be absent since birth in his hand and wrist.

After approximately 5 h of serial examinations, as well as extensive discussion with the orthopaedic, plastic surgery, neonatology, and pediatric anesthesia teams, as well as the infant’s parents, the decision was made to perform decompressive fasciotomy of the forearm, hand, and carpal tunnel to enable limb salvage. Fasciotomy was performed at approximately 6 h after birth (Figure 2A and B). The infant was monitored closely throughout the following days. The appearance of the left upper extremity gradually improved. On day three of life, the forearm and hand had improved markedly in color and appeared well vascularized (Figure 3A and B). Doppler signals were present throughout, from the brachial artery to each digit. The fasciotomy wounds were allowed to heal by secondary intention, and a custom splint was applied with the wrist in neutral position and the fingers extended. No signs of infection or ongoing ischemia were present. The wounds were well healed by six weeks of age (Figure 4A and B). At the follow-up visit at four months of age, the wounds were fully healed (Figure 4C). The patient had full passive range of motion of the wrist and fingers. He was noted to have slight flexion and extension of the ring and small fingers, but otherwise minimal active motion of the wrist or hand at this point. He had active motion of the shoulder and elbow. The patient will continue receiving occupational therapy, as well as splinting and scar massage to prevent/minimize contractures. Multidisciplinary teams will continue following his progress and recovery closely.

## Discussion

In a series of 24 patients, Ragland et al. [4] reported no association between neonatal or maternal conditions and compartment syndrome. No cases of oligohydramnios, uterine abnormalities, or amniotic disruption sequence were observed; however, one case of polycystic kidneys was reported. Notably, all patients had skin lesions of various sizes [4]. Agrawal et al. [8] reported that nearly 20% of 50 patients with neonatal ischemic contracture were born prematurely. In many cases, the initial presentation and earliest sign of NCS is a superficial sentinel skin lesion [4]. Such lesions vary in appearance, ranging from skin discoloration, bullae formation, desquamation, to necrosis [8]. Progression of NCS may result in muscular and neuronal ischemia, contractures, and limb-growth disturbances [1, 4]. Decompressive fasciotomy is necessary to limit the risk of poor outcomes and long-term function [3]. Despite early intervention, long-term outcomes are poor in many cases [3]. Given the rarity of the condition and the potentially devastating outcomes, prompt multidisciplinary evaluation is critical.

We describe a case of NCS in a newborn male with lower urinary tract obstruction, delivered by cesarean section at 35 weeks’ gestation. The etiology of NCS in this case was hypothesized to be due to compression of uterine wall on fetus in setting of oligohydramnios. Prompt evaluation was performed by a multidisciplinary team (including specialists in neonatology, anesthesia, orthopaedics, and plastic surgery), enabling diagnosis and appropriate treatment. This case highlights the need for increased awareness of the risk developing compartment syndrome in utero in infants born with congenital kidney disease and oligohydramnios.

## Take-home messages of lessons learned

Neonatal compartment syndrome is a rare condition that requires early diagnosis to enable effective treatment and optimize functional outcomes. We report a case of neonatal compartment syndrome diagnosed largely because of the appearance of the skin lesions on the forearm and treated within five to 6 h after birth. The early outcomes of prompt surgical intervention are reported. Prompt diagnosis and multidisciplinary evaluation are critical. This case highlights the need for increased awareness of the risk developing compartment syndrome in utero as part of the rare sequalae in infants with congenital anomalies of kidney and urinary tract.
